# Managing dementia care during COVID-19 pandemic: caregivers’ experiences in Odisha, India

**DOI:** 10.1017/S1463423622000664

**Published:** 2023-05-25

**Authors:** Pranab Mahapatra, Krushna Chandra Sahoo, Shyama Desaraju, Binapani Nath, Sanghamitra Pati

**Affiliations:** 1 Department of Psychiatry, Kalinga Institute of Medical Sciences, Bhubaneswar, Odisha 751024, India; 2 Regional Medical Research Centre, Indian Council of Medical Research, Bhubaneswar, Odisha 751023, India

**Keywords:** caregiver perspective, dementia, India, LMIC, multimorbidity care, Pandemic, qualitative, SARS Cov 2

## Abstract

**Aim:**

The present study explored the family caregivers’ perspectives and elicited their experience while managing dementia care during the COVID-19 pandemic in Odisha, India.

**Background:**

The onset of the COVID-19 pandemic has diverted the attention of health systems away from chronic disease management and health services delivery. Psychiatric care particularly for dementia and the elderly is found to be more compromised in such situation.

**Methods:**

We adopted an inductive phenomenological approach to garner key insights into the care continuity for people living with dementia in the context of the COVID-19 pandemic. Telephonic in-depth interviews (IDIs) were carried out with 17 immediate caregivers. All IDIs were digitally recorded, transcribed, and analysed using a thematic approach.

**Findings:**

Caregivers did not perceive dementia as an overwhelming challenge; instead viewed it as a part of the ageing process. Caring for dementia was being done by family members as a collective responsibility with task-sharing. The caregivers primarily relied on their usual physician for the continuity of dementia care and took utmost precautions to prevent exposure to COVID-19 risk. However, they found it more challenging to ensure adequate care for the multiple illnesses (multimorbidity) coexisting with dementia. Towards this, they adopted all possible measures to keep the chronic conditions under control, lest the vulnerability to COVID-19 infection might heighten. The fear of visiting a hospital, prevailing restrictions in mobility, and diverted attention of health systems to pandemic containment created impediments towards maintaining multimorbidity care. The support of local administration, neighbourhood pharmacy and diagnostic laboratories and teleconsultation with the physicians were vital for care continuity. Caregivers adapted by reducing or deferring physical consultation and seeking treatment via telephonic advice of the treating physicians. Our findings suggest leveraging digitally enabled health care technology and augmenting caregiver activation for home-based dementia care to cruise through any similar catastrophic situations.

## Background

Globally, over 50 million people are living with dementia, with about 10 million new cases per year (WHO, 2020) – most of them reside in low- and middle-income countries (LMICs) (Ferri and Jacob, 2017; Rodriguez and Roehr, 2020). India, the largest LMIC democracy, is witnessing an increasing prevalence of dementia, with around 2.5% (ranging from 1% to 6%) elderly living with the condition (Das *et al.*, 2012; Vaitheswaran *et al.*, 2020). Dementia, a disorder in which memory, thinking, behaviour, and day-to-day activities deteriorate, is one of the leading causes of disability and dependency among the geriatric population worldwide (Krysinska *et al.*, 2017; Woodbridge *et al.*, 2018; Livingston *et al.*, 2020). Treatment and rehabilitation of persons with dementia is a significant concern given its substantial physical, psychosocial, and economic impact on them and their caregivers, family, and society (Killin *et al.*, 2016; Karr *et al.*, 2018; Peters *et al.*, 2019; Lamech *et al.*, 2019).

The elderly are more vulnerable to disasters or emergencies than the younger population (UN, 2015); this has also been observed during the COVID-19 pandemic (Alonso-Lana *et al.*, 2020; Hariyanto *et al.*, 2020). Moreover, loneliness is highest among these age groups (Reher and Requena, 2018; Esteve *et al.*, 2020). Many of them had no face-to-face interaction with their family members during the mandatory stay-at-home situation (Banerjee *et al.*, 2020; Ferini-Strambi and Salsone, 2020). As a result, COVID-19 has affected their everyday life, health care, and emerging health needs (Alonso-Lana *et al.*, 2020; Bolt *et al.*, 2020; Giebel *et al.*, 2020).

The onset of the COVID-19 pandemic has diverted the focus of health systems towards containing the spread of infection, thus concomitantly resulting in disruptions in health services delivery for chronic diseases to a large extent. Our community-based studies on the management of non-communicable diseases (NCD) during COVID-19 lockdown have revealed that people of higher age, having mental illness had significantly more significant care challenges (Pati et al., 2020b; Sahoo *et al.*, 2020). The presence of dementia and the impact of the COVID-19 pandemic pose considerable concerns for the elderly.

Global evidence indicates that people with dementia have difficulty recognising and adhering to COVID-19-related precautions such as wearing masks, maintaining physical distance, hand hygiene, and the necessary steps for self-quarantine (Bukhman *et al.*, 2020; Holmes *et al.*, 2020), which can cause difficulty in managing them during the stay-at-home time (Bianchetti *et al.*, 2020; Canevelli *et al.*, 2020; O’Shea 2020; Wang *et al.*, 2020). Hence, it is necessary to understand the experiences of caregivers in managing older adults with dementia during the COVID-19 pandemic and assess how they continued to provide care under the prevailing new-normal situation. To date, little research has been carried out in this area; we undertook a qualitative study to explore caregivers’ perspectives and elicited their experiences with managing dementia care during the COVID-19 pandemic in Odisha, India. The findings garnered could provide critical insights into managing psychiatric and geriatric care during similar catastrophic situations.

## Methods

### Study design, settings, and participants

We purposively selected Bhubaneswar, the capital city of Odisha, for our study. This city is located in Khurda, one of the first and most affected COVID-19 districts in the state (Govt. of Odisha, 2020). From our previous cohort database of psychiatric multimorbidity patients, we identified 22 older adults with dementia residing in Bhubaneswar (Pati et al., 2020b).

We reached out to 20 family members of individuals with dementia over the telephone, briefed them regarding the study objective, and solicited their consent to participate. From these, 17 individuals, either a family member or an immediate caregiver, agreed to be interviewed. We adopted a phenomenological approach to understand the care delivery process for people with dementia during the COVID-19 pandemic. A semi-structured open-ended guide (Table 1) was developed through a consultative and iterative process. A total of 17 in-depth telephone interviews (IDIs) were performed, one interviewee for each patient with dementia. There were no repeat interviews.

**Table 1.** In-depth interviews guide

1	Request and capture the patients’ demographic details, home environment, and support systems.
2	When was the dementia diagnosed? What other illnesses are there?
3	How much is he able to manage his everyday activities? What is the assistance he needs for day-to-day activities?
4	What were their daily health-related activities? Like diet, exercise, meditation, or any other? (for Dementia as well as for comorbidities if any)
5	How were their health issues managed before the pandemic? Routine and emergency physician consultations, Interventions like daycare, chemotherapy, physiotherapy etc. What support did they need to manage their care?
6	What were the apprehensions towards the COVID-19 pandemic?
	What precautions did they take?
7	How did the pandemic affect healthcare? During this period, explore their consultations, emergency preparedness, and other needs like chemotherapy, physiotherapy, and daily health activities.
8	How did they manage health issues– the consultations, allied services, interventions and investigations, procuring medications? To what extent were they able to self-manage their illnesses?
9	How were other elements like transport, finance and getting help from others managed?
10	What are your suggestions to improve healthcare access in similar situations?

### Data collection procedure, data management, and analysis

We conducted the IDIs in June 2020, immediately following the first phase of unlocking. The Indian government ordered a nationwide lockdown from March 24 to May 31, 2020. All IDIs were carried out by the first author (PM), with clinical psychiatry background having experience in community-based research and native study settings. Each interview spanned from 20 to 35 min. The interviews were digitally recorded, transcribed, and translated into English. The authors – SD and BN transcribed and translated the interviews. The authors – PM and KCS coded the data and prepared the coding tree, and SP reviewed the coding.

The data were analysed by thematic approach, and an inductive method to analysis was perused. The findings were extended to an interpretive level to gain new insights into the above phenomenon. First, the meaning units were outlined from the transcript of the interview and coded. We did open coding and then first cycle coding by using – process, concept, emotion, and values coding. The related codes were next grouped together (axial coding); finally, the theme was derived (Miles *et al.*, 2014). The coding of data based on the identified themes and categories was done with MAXQDA software (MAXQDA Analytics Pro 2020, VERBI GmbH, Berlin, Germany). After preliminary analysis, the findings were debriefed with four participants for member check. The Consolidated Criteria for the Reporting of Qualitative Research (COREQ) guideline was used to report the study (Booth *et al.*, 2014). All authors were involved in the analysis. The authors’ diverse educational and professional background, along with their experience in public health research, facilitated a broadened interpretation of the findings.

### Ethical considerations

We obtained ethical clearance from the Institutional Ethical Committee of the ICMR-Regional Medical Research Centre, Bhubaneswar (ICMR-RMRCB/IHEC-2020/027). The study adhered to the Declaration of Helsinki and later amendments. Each interviewee was given a unique identification number for anonymity. Telephonic consent was obtained before proceeding with the interview, and the IDIs were audio-recorded with prior permission. All necessary measures were taken to ensure the privacy and confidentiality of the data.

## Findings

All of the persons with dementia resided with their families. Their ages ranged from 58 to 84 years old (mean 67, SD 6.9), and their average duration of dementia is 4 years. Ten of the 17 were males, with the remaining seven being females. They were all from urban or peri-urban areas, with nine hailing from higher socio-economic groups and eight from lower socio-economic groups. Most of the immediate caregivers were family members, primarily females – a wife, daughter-in-law, or daughter. Three families in urban areas had part-time home nurses as additional support because the family caregivers were unable to stay with them always.

Two major themes emerged: (1) Managing dementia care as usual and (2) Managing dementia care during the COVID-19 pandemic. The coding tree derived from the analysis is depicted in Table 2.

**Table 2.** Detailed coding tree

Themes	Managing dementia care as usual			Managing dementia care during COVID-19 pandemic		
Categories	Routine care for dementia	Care for coexisting illness	Care delivery perspective and challenges	Protecting against COVID-19	Caring for coexisting illness	Care continuity experiences
Codes	Adjusted to routine life	Better access to healthcare	Access to diagnostic facility	Barest minimum contact with outsiders	Avoid hospitals	Appreciative government’s initiatives
	Disruptive needing medical assistance	Coexisting multiple illnesses	Availability of physician	Increased family accompanies	Deferred routine consultations	Avoided physical presence
	Impaired resilience	Emergency needs	Avoiding marriage plans	Isolated the patient	Difficult to seek care	Difficult to consult a regular physician
	Increased care burden	Multiple conditions	Collectively involved in the patient’s care	Media channels	Fear of an impending emergency	Doctor guided over the phone
	Less fear of infections	Need multiple hospital visits	Duty to look after	Meticulous precautions	Home-based treatment	Enough time to provide care
	Poor self-management	Other chronic illnesses	Hired part time home nurses	Needs vigilance	Need escorts	Healthcare support
	Unable to decide daily activities	Periodically consult	Less time to provide care	Perceived vulnerability	Need multiple hospital visits	Support of local administration
		Physiotherapy	Never left unattended	Self-protection	Telemedicine facility	Support of neighbourhood pharmacy and laboratories
		Regular laboratory investigations	Shared roles and responsibilities	Social distancing		
				Wearing masks		

### Theme 1: managing dementia care as usual

*Routine care for dementia:* Caregivers reported that persons with dementia had difficulty carrying out daily activities like personal hygiene, bathing, taking their food, or walking. Even they could not remember to take prescribed medications, nor could they follow physicians’ advice. All interviewees described how they took elaborate and meticulous care of all daily activities and routine health chores of the patients ‘*as they do for a child’*. They also tried to make the patients watch television, engage in conversations or accompany them for a walk whenever possible. Few recalled stray instances when the patient would become agitated and disruptive, needing medical assistance. Most caregivers felt that dementia was not a major problem to manage as they have got used to it, with few disagreeing with the diagnosis, saying ‘he can remember many things, even the oldest of memories’.

*“He used to do his work, but for the last 7–8 months, we do all his work, assist in toilet and bathroom, medicine, food. The way a parent does for their child”*. [Daughter-in-law, 29 years]

*“He wakes up in the middle of the night and leaves from the house, and does not recognise his own house. We have to closely guard him like a child”*. [Daughter-in-law, 36 years]

*Care for coexisting illness*: Almost all of the carers reported that the patients had other chronic illnesses in addition to dementia. For these ailments, they periodically consult various clinicians at different facilities. They use to get regular laboratory investigations, treatment, and other procedures like physiotherapy to keep the illness in control. Some of the carers reported emergency needs like one patient had to undergo a daycare procedure, and two were admitted to the hospital for these coexisting conditions.

Most family caregivers preferred to consult the same physicians, either at private clinics or hospitals. Owing to multiple constraints, they had to schedule an appointment, accompany them and arrange transport accordingly. Coordinating between these consultations needed more attention than for dementia. Many caregivers believed that more than dementia, coexisting multiple illnesses were challenging to manage.

“She has diabetes for at least five years. We get her tested every month and medicines are given. She repeatedly suffers from urinary incontinence and psychiatric problems for which she gets referred to another hospitals”. [Daughter-in-law, 34 years]

*Care delivery perspective and challenges:* Almost all family caregivers considered it their duty to look after a family member with dementia. They did not hire a caregiver as they felt that ‘an outsider cannot take similar care’ and the family can do a better job since they know the person’s moods and needs very well. They explained that all family members were collectively involved in the patient’s care with shared roles and responsibilities. Female family members, wife, daughter-in-law, or daughter, were the immediate caregivers managing daily activities like personal hygiene, feeding, and medications, while the male members usually took up the responsibility of arranging physician consultations and treatment.

*“We need to be mentally strong to manage the situation. Since we are living in a joint family that is an advantage. My brother and myself share the work following a schedule, we prioritise his care. Every day, we clean, bath and dress up him. We take care of his breakfast and medicines”*. [Daughter 25 years]

Caregivers from families with fewer members reported giving a major share of their time to patient care, leaving less time for their own. Some of these family caregivers took the assistance of household help for patient care, and three of them had hired part-time home nurses. In two instances, where no active family members are available for care and the children of persons with dementia made crucial personal choices, like avoiding marriage and career plans to devote full time for the patient. All the family members saw to it that their patient was never left unattended.

“We make it a point to never leave him alone. We do rotation or shifts and be with him 24/7 h”. [Daughter-in-law, 29 years]

### Theme 2: managing dementia care during the COVID-19 pandemic

*Protecting against COVID-19*: Many of the caregivers told that they came to know from media channels that elderly with coexisting chronic disease have a very high risk for COVID-19 infection, which may turn severe. Accordingly, they took several precautionary measures to protect their patients from any possible risk of exposure. Most interviewees told that they isolated the patient and allowed barest minimum contact with outsiders. Those family members going outside, or any outsiders visiting needed to sanitise themselves, wear protection and maintain a distance while meeting the patient.

Caregivers explained that as their patients could not follow appropriate COVID-19 preventive measures like wearing masks, social distancing, and other self-protection on their own, hence the families were always vigilant, keeping a watch on them. Even, few families withheld all domestic help’s services for a while when the spread of infection was very high across the town. Those who had hired part-time nurses took meticulous precautions, choosing people known to them and preferring those who could maintain proper sanitisation. One of the participants mentioned that hired caregivers were tested for COVID-19 by their agencies during recruitment.

*Caring for coexisting illness:* The interviewees viewed that age and other illnesses made their patient susceptible to COVID-19 infection. Speaking about her mother, one caregiver explained that she has a heart problem, high blood pressure, mental issues, and overweight that make her susceptible; thus, they avoid hospitals. They viewed all places outside house with a tint of suspicion. Hospitals and public transports were perceived as potential sources of infection. Many confided that they were apprehensive of COVID-19 circulating in the air and might infect their patients while visiting a clinic or hospital. Some of the caregivers voiced concern that their patient might not survive if they get infected with COVID-19.

*“We try to avoid public vehicles and ambulance. Who knows, they might be harboring the infection as COVID patients are coming in the ambulance”* [Daughter, 27 years]

Caregivers reported that they deferred routine consultations and tried their best to manage at home by seeking telephonic advice. They also did not carry out regular investigations and some procedures like physiotherapy and those involving daycare. Some said that they approached hospitals when it was unavoidable. Some of the caregivers preferred to go to private clinics because of less COVID fear as it is not that crowded.

Though caregivers avoided routine consultations, there was always the fear of an impending emergency. Some kept in touch with their physicians through phone, and a few needed emergency care. Many continued the same treatment when their patients were without any active symptoms. A few of them consulted “in absentia”, in which one of the relatives physically consulted their physician to share the patient’s complaints along with the past prescriptions and laboratory reports. On one occasion, the family caregivers suspected COVID infection in the member with dementia when he showed fever. They feared that he would be taken to designated COVID hospitals and treated in isolation. Hence they treated him at home, without consulting a physician. For investigations, they took the help of local laboratories by scheduling a home visit and sample collection. Most caregivers reported using online and informal telephonic consultation as an alternative to physical consultation.

“Unfortunately, the doctor treating my mother himself got COVID-19. So we had to consult another physician. It was difficult for us to explain as he had not seen my mother before and did not know her history of illness”. [Wife 52 years]

*“The doctor responded every time I called. That was a great help to me. That gives much moral support not only to the patient but also family members”*. [Daughter-in-law, 30 years]

The caregivers said that hospital services had changed due to the COVID-19 pandemic. All patients needed to be screened for COVID-19 on arrival at a hospital. One of them said that their patient had to wait outside for quite some time, for which they were worried that his condition would deteriorate. Some also apprehensive about their patients getting infected while waiting for admission. Their major fear was their patient would be isolated if found positive for COVID-19 and then succumb as they are frail and unable to fend for themselves. Mostly their physicians encouraged home-based treatment and advised admission only for the severely ill.

“If we get infected, we can survive; but if she gets infected, then it would be very difficult. Rarely, I use to go to outside, and I always wear masks and gloves. After coming back, I take a bath. Still, we are scared of getting the infection, and she is not even wearing the mask; she is removing and throwing it”. [Daughter, 25 years]

*Care continuity experiences*: Most of the respondents adapted to the changed pandemic scenario. One of the caregivers considered ‘COVID-19 is the best thing and the worst thing that has happened, amidst all adversities, they could give more time to their patients. They reported that friends and relatives were in constant touch through the phone even though they avoided physical presence. Those in quarantine took the help of neighbours for buying medicines and their daily needs. All of them agreed that government agencies like police and ambulance services were available for any healthcare support. Most of the caregivers spoke about reaching out to their regular physician for help as they knew about the patient and often guided them over the phone. They found these informal telephone calls reassuring. Whenever they visited a hospital with health issues, they took utmost precautions to avoid getting infected. All caregivers described how they were taking double efforts for self-protection and adopting all possible ways not to bring or transmit COVID-19 infection to their patient. The caregivers appreciated the government’s initiatives to allay the fear of COVID among people. Fear reduced further over time with treatment protocols and medicines improving the recovery rate of COVID-19 patients.

“The doctor made video calls to my mother, and she was delighted as she was able to see him…that gives much moral support to the patient and family members of the patient”. [Wife, 55 years]

“We had strong COVID fear in our mind, but the government ensured that there is nothing to panic”. [Daughter-in-law, 35 years]

**Figure 1.**
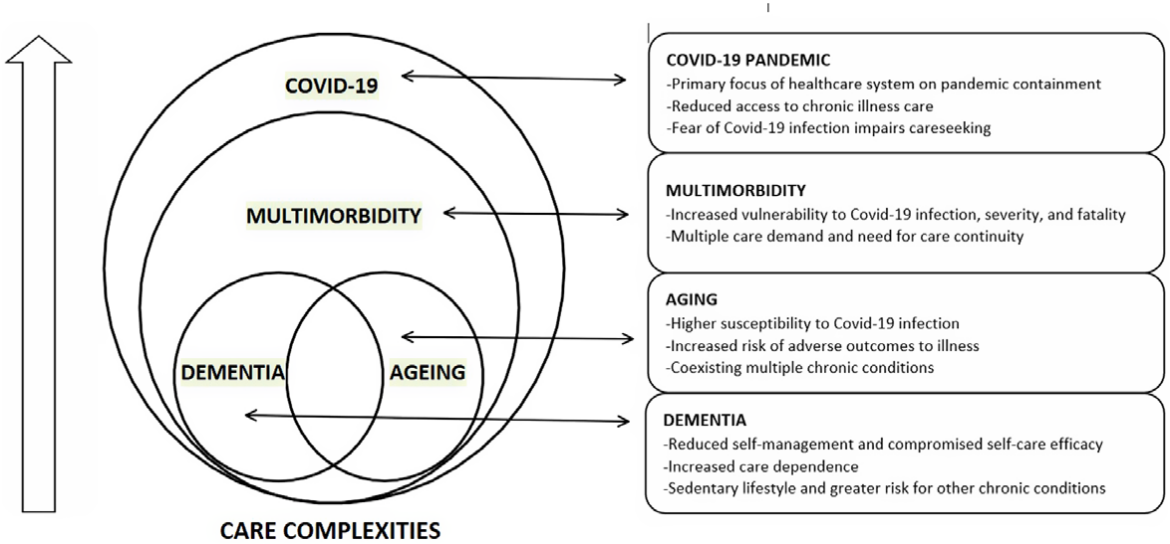
Conceptual framework on complexities for dementia care needs during COVID-19 pandemic

In the context of the COVID-19 pandemic, care delivery became more complex for people with dementia. Figure 1 depicts the conceptual framework of the underlying care complexities while managing dementia. Persons with dementia have impaired self-management and self-care efficacy, thus leading to increased care dependency. Besides, they have a sedentary lifestyle. Ageing is associated with increased susceptibility to COVID-19 and an increased risk of adverse health outcomes. Furthermore, the presence of multimorbidity increases the risk of COVID-19 infection, severity, and death, necessitating multiple care needs and affects care continuity. During the pandemic, the health system’s primary focus was on COVID-19 care, limiting access to care for other chronic diseases.

## Discussion

Caregivers did not perceive dementia as a separate challenge; instead, they attributed it to the process of ageing. Family members mainly carried out dementia care as a collective responsibility with sharing of the task. The caregivers primarily relied on their usual physician for the continuity of treatment and took utmost precautions to protect against exposure to COVID-19. Their primary challenge was to ensure adequate care for other coexisting chronic illnesses (multimorbidity). They adopted all possible measures to keep these chronic conditions under control. The prevailing restriction in mobility, fear of going to the hospital, and diverted focus of the health system to pandemic management posed impediments towards ensuring treatment continuity. The support of local administration, neighbourhood pharmacy, diagnostic laboratories, and teleconsultation with the physicians were the key enablers of treatment. Regular consultations were deferred and treatment continued by telephonic advice of the treating physician.

Evidence worldwide has shown that older people are at increased risk from COVID-19 due to their compromised immunity, which reduces the resistance to infection and increases recovery time (Rahman *et al.*, 2020). Elderly fatalities were found to be higher than younger people (Cerasoli, 2020). Moreover, in dementia, the person concerned has low self-care and self-efficacy, resulting in lowered personal hygiene, diet, and physical activity and increased care dependence. Declining cognitive impairment is associated with loss of other Instrumental Activities of Daily Living (IADLs), such as managing medications and participation in the treatment process (Mlinaca and Feng, 2016). Along with ageing and dementia, the presence of multiple coexisting illnesses, i.e. multimorbidity, posits significant care challenges (Kessler and Bowen, 2020; Pati *et al.,* 2020c), accentuated in the COVID-19 context. Restricted movement and reduced focus of the health system for chronic care management reduce care access during the pandemic. Additionally, the number of hospital visits was lesser than usual since the hospitals were perceived as a potential source of infection and commuting to the health care facility increased the risk of exposure to COVID-19.

The presence of multimorbidity and multiple points of care increases the risk of COVID-19 infection, its severity and fatality, and increases healthcare needs (Kessler and Bowen, 2020). It also highlights the importance of care for patients with dementia with other ageing related conditions, which has been already reported worldwide (Pati *et al.,* 2015b; Kshatri *et al.*, 2020). Recent research conducted by Pati et al., in Odisha, India, on multimorbidity and its outcomes among patients in psychiatric care settings found that half of the psychiatric outpatients had multimorbidity (Pati *et al.,* 2020b). The relative odds for multiple morbidities is 6.6 times higher for patients over 60 years of age (Pati *et al.,* 2020b). Multimorbidity, along with psychiatric disorders, lead to a substantial rise in healthcare expenditure (Pati *et al.*, 2014, 2015a, 2019a, 2020b) and complicates the already limited access to treatment (Pati *et al.,* 2015a, 2019b; Mahapatra *et al.*, 2019; Jena *et al.*, 2020). Psychiatric multimorbidity, including dementia, is an emerging global concern among the elderly.

Healthcare is socially shaped and culturally construed (Kleinman, 1978). The living environment of the elderly and the socio-cultural fabric encompassing various strategies adopted in communities with diverse cultural and historical traditions have a significant role in dementia care (Lamech *et al.*, 2019). Empathy and person-centred care should be considered necessary along with family care (Sommerlad *et al.*, 2018; Van Dalen *et al.*, 2018). Besides, an extensive assessment of care arrangements among dementia caregivers in a multicentric study in India, China and South-East Asia, Latin America, and the Caribbean and Africa showed that most caregivers were women, living with the person with dementia in the extended family or household (Prince, 2004). They concluded that larger households were associated with lower caregiver strain, wherein the caregiver was co-resident (Prince, 2004), similar to our findings. In India, mainly, care delivery is family-centric. The caregivers are genuinely concerned for the elderly living with dementia; they care for them ‘as a child’ and do not consider them a burden. Besides, in the extended family, they make it a practice to keep a constant vigil on the patient on rotation or in shifts. Our study revealed that task-sharing rather than task-shifting have strong relevance, especially for dementia care among the elderly (Pati et al. 2019b; Jena *et al.*, 2020). Task shifting is a delegation process in which tasks are moved, along with workforce reorganisation. Task sharing allows performing tasks and procedures that would usually share by all members. Also, there is ample evidence of the underlying concept of ontological security among people caring for mental illness by saying, ‘there is no place like their own home’ (Savla *et al.*, 2020). The ontological protection indicators were constancy, everyday habits, anonymity, and a stable base for identity building (Padgett, 2007).

The older adults with COVID-19 infection appear to have atypical symptoms, ranging from afebrile with non-respiratory symptoms such as delirium or isolated functional decay to being afebrile with no apparent physical symptoms (Emami *et al.* 2020; Hariyanto *et al.*, 2020). Therefore, medication management for the elderly with dementia is mainly provided by family caregivers (Lim *et al.*, 2020). In our study, the neighbourhood pharmacy and laboratory provided door-step services during the pandemic. There is also evidence of interventions to support cognitive issues, such as communication and awareness of prescribed medicines’ indications (Lim and Sharmeen, 2018; Sinclair and Abdelhafiz, 2020). A previous study in India suggested a pragmatic, multi-layered approach that included institutional reform, enhanced understanding, and technological usage is crucial for dementia caregivers during the pandemic (Vaitheswaran *et al.*, 2020).

The change of health care providers during the pandemic was one of the challenges experienced among the participants. The doctor–patient relationship is considered a key element in healthcare practice, especially in telemedicine (Mahajan *et al.*, 2020; Kemp *et al.,* 2020). There is an establishment of a therapeutic relationship when a physician responds to a patient’s medical needs, such as routine check-up, diagnosis, and care in an appropriate manner (Gomes *et al.*, 2020). Although, access to technology and health care is a significant concern for many who cannot afford private services in India. However, the doctor–patient relationship plays an important role (Ananthakrishnan and Singh, 2020; Mahajan *et al.*, 2020). It is essential to maintain a therapeutic alliance between care providers, caregivers, and patients for appropriate treatment.

### Trustworthiness and reflectivity

In order to enhance the trustworthiness of the study, we pursued research and source triangulation. Given our diverse academic and professional backgrounds, we interpreted the study results with broad perspectives; participants came from different socio-economic backgrounds, with both male and female participants from different settings. All of the interviews were conducted by the first author, who was a treating clinician. This was because most of the caregivers preferred to be interviewed by their usual care provider. These interviews were mostly explorative in approach, adhering to the pre-designed interview guide.

In contrast, during routine psychiatric consultation, diagnostic interviews usually probe and eliminate possibilities, thus funnelling towards a diagnosis. Treatment-related preunderstanding was bracketed during the interview; for example, no attempt was made to explain the illness during the interview. Instead, their queries were clarified after the interviews. The responses were discussed in a non-judgemental way. We adopted cross-checking procedures during transcript analysis – vernacular (Odia) and English transcripts were used in tandem and cross-checked during the coding process to garner the transcript’s in-depth context. During data collection and analysis, the researchers’ pre-understanding was bundled. While we conducted a study in Odisha, the results may be helpful in other similar LMIC settings. The study’s limitation is non-probability sampling, and we identified participants through contact with clinicians, which is not representative and not generalisable.

## Conclusions

Dementia care in India is more home-based, with the close involvement of family members. Infection control and prevention measures against COVID-19 for these vulnerable group is to be reinforced. In any catastrophic situation, the provision of chronic disease management services should be carried out to prevent major care disruption. Managing both physical and mental care among the elderly needs to be considered while preparing a pandemic and post-pandemic exit plan. Our findings suggest augmenting digital healthcare and community activation for home-based care for elders with dementia.
